# P050 Neurosyphilis - an unexpected mimic of giant cell arteritis

**DOI:** 10.1093/rap/rkag113.080

**Published:** 2026-11-03

**Authors:** Isabella Patterson, Ceril Rhys-Dillon

**Affiliations:** Rheumatology, Cwm Taf Morgannwg, Cardiff, United Kingdom; Rheumatology, Cwm Taf Morgannwg, Cardiff, United Kingdom

## Abstract

**Introduction:**

Syphilis, an infectious disease caused by the spirochete bacterium *Treponema pallidum*, has continued to increase in prevalence in the UK in recent years. Ocular syphilis is a manifestation of neurosyphilis and can occur at any stage of infection. Symptoms often include visual blurring, but there is a risk of complete vision loss if untreated. Visual changes are seen in rheumatology primarily in the context of giant cell arteritis (GCA), a large-vessel vasculitis affecting the older population. This case highlights ocular syphilis as a mimic for GCA and the importance of a good history when reviewing those with suspected GCA.

**Case description:**

A 70-year-old Caucasian male with a background of gout, TIA, GORD and right eye congenital cataract presented to ophthalmology with a three-day history of reduced vision in the left eye. Ophthalmic assessment identified reduced visual acuity at 6/36 with normal fundoscopy. Initial investigations showed CRP 39, ESR 126 and CT head normal. Given loss of vision and raised ESR, GCA was considered, and he was treated with IV methylprednisolone 1g for three days. He was referred to rheumatology outpatients on daily prednisolone 60mg.

Subsequent review by rheumatology revealed no history of constitutional symptoms, jaw claudication or headache with no features concerning for vasculitis or connective tissue disease. GCA was therefore considered unlikely. Further questioning identified multiple episodes of unprotected sexual intercourse with sex workers during recent travel in the Philippines. Full sexually transmitted infection screening was performed. Chlamydia, gonorrhoea and toxoplasma tested negative, but results were positive for syphilis TPHA (>1:10240) and RPR (1:128), indicative of active syphilis infection.

The patient was referred to the genitourinary (GU) service where IM benzylpenicillin was administered. The case was discussed in a virtual MDT involving infectious diseases, ophthalmology, rheumatology and GU medicine, which concluded further treatment for ocular syphilis with a 14-day antibiotic course was necessary.

Three months prior, he presented to his GP with burning leg pain. Low iron and a subsequent positive FIT test triggered a CT TAP. The CT demonstrated multifocal patchy areas of consolidation and segmental collapse, widespread small nodular changes with a small right paraoesophageal lymph node. Subsequent discussion at respiratory MDT identified pulmonary syphilis as the likely cause, which is to be monitored with interval scans.

Further telephone review identified that he feels much better with full sight resolution. No ongoing inflammatory or infective symptoms are present, and he has since returned to the Philippines.

**Discussion:**

Both GCA and ocular syphilis can be diagnostically challenging, which can lead to delays in diagnosis. However, with rates of syphilis continuing to rise in the UK, this case highlights the need for suspicion of syphilis in those with unexplained ophthalmic symptoms.

Early neurosyphilis, including ocular disease, is rare, occurring in only 1-2% of those with secondary syphilis and potentially fewer with primary disease. A previous case report of ocular syphilis as a GCA mimic included constitutional symptoms with no initial visual symptoms, contrasting to this case. The variation in presentation of ocular syphilis provides further diagnostic complexity but emphasises a sexual health history is essential.

The British Society for Rheumatology GCA guidelines are currently under review. Temporal artery ultrasound and biopsy are currently gold standard investigations and negative confirmatory testing may have refined the differential diagnosis earlier. Yet the Southend GCA probability score was 10 and syphilis was diagnosed. Sex tourism is common and prevalent in certain locations; it was this attention to detail that enabled the correct diagnosis.

The rheumatology team were not involved in the care of this patient until he was seen in outpatient clinic. Involvement of rheumatology during inpatient admission may have been valuable in questioning the diagnosis of GCA earlier. Early diagnosis is essential for harm reduction, allowing for sight preservation and reduced morbidity. Additionally, unnecessary steroid exposure can be avoided.

A challenge in the management of this patient was the split of care between multiple teams. The GU department uses a different patient record system, which led to delayed communication; this was addressed through the virtual MDT. An additional diagnostic challenge was the patient’s existing right eye blindness, which led to the ocular syphilis presenting unilaterally rather than the more common bilateral presentation.

**Key learning points:**

• This case demonstrates the importance of a detailed and inquisitive history, including sexual health, which may not be routine practice in a GCA-aged population. Rheumatologists are often faced with diagnostic uncertainty, but it was the detailed travel and sexual history that ultimately led to the diagnosis of syphilis in the patient and prevented further sight loss and ongoing syphilis transmission.

• The use of the MDT was essential in recognition and diagnosis for this patient. Ongoing collaboration and interdisciplinary education between these specialities will enable more accurate and efficient diagnoses of GCA and its mimics going forward.

• With the GCA guidelines currently under revision, it raises the question as to whether ocular syphilis should be a listed mimic and sexual health should be recommended as part of the GCA history.

